# Probiotic–Vaccine Synergy in Fish Aquaculture: Exploring Microbiome-Immune Interactions for Enhanced Vaccine Efficacy

**DOI:** 10.3390/biology14060629

**Published:** 2025-05-29

**Authors:** Muhammad Tayyab, Waqar Islam, Waqas Waqas, Yueling Zhang

**Affiliations:** 1 Guangdong Provincial Key Laboratory of Marine Biology, Institute of Marine Sciences, Shantou University, Shantou 515063, China; 2 Xinjiang Key Laboratory of Desert Plant Roots Ecology and Vegetation Restoration, Xinjiang Institute of Ecology and Geography, Chinese Academy of Sciences, Urumqi 830011, China; waqarislam@ms.xjb.ac.cn

**Keywords:** probiotics, vaccines, aquaculture, mucosal immunity, microbiome modulation, synbiotics, immune priming, sustainable aquaculture, one health, antibiotic reduction

## Abstract

The rapid expansion of aquaculture is vital for global food security, yet it faces persistent threats from disease outbreaks, vaccine inefficacy, and antibiotic overuse, all of which undermine sustainability. Conventional vaccines often fail to induce robust mucosal immunity, spurring interest in probiotics as adjuvants to enhance immunogenicity. Probiotics such as *Bacillus subtilis* and *Lactobacillus casei* modulate fish microbiomes, fortify mucosal barriers, and activate innate immune responses via mechanisms including Toll-like receptor signaling and cytokine production. These actions prime the host environment for prolonged adaptive immunity, improving antigen uptake and pathogen clearance. Experimental advances—such as *Bacillus subtilis*-engineered spores increasing survival rates to 86% in *Vibrio anguillarum*-challenged European seabass—demonstrate the potential of this synergy. Innovations in delivery systems, including chitosan–alginate microcapsules and synbiotic formulations, further address oral vaccine degradation, enhancing practicality. Probiotics also suppress pathogens while enriching beneficial gut taxa, amplifying mucosal IgA and systemic IgM responses. However, challenges such as strain-specific variability, environmental dependencies, and unresolved ecological risks persist. Optimizing host-specific probiotics and advancing multi-omics research is critical to unlocking this synergy fully. Integrating probiotic mechanisms with vaccine design offers a pathway toward antibiotic-free aquaculture, aligning with One Health principles. Realizing this vision demands interdisciplinary collaboration to standardize protocols, validate field efficacy, and align policies with ecological sustainability. Probiotic–vaccine strategies represent not merely a scientific advance but an essential evolution for resilient, ecologically balanced aquaculture systems.

## Simple Summary

Global aquaculture is under pressure from disease outbreaks, excessive antibiotic use, and uneven vaccine efficacy, jeopardizing food security. This review explores how probiotics—beneficial bacteria such as *Bacillus* and *Lactobacillus*—work together with vaccines to boost fish immunity. Probiotics enhance mucosal defenses, suppress pathogens like *Vibrio*, and improve vaccine performance through methods such as microencapsulation. Trials have shown up to 86% survival in pathogen-challenged fish. Despite promising results, issues like strain consistency and environmental safety need more study. Adopting probiotic–vaccine strategies can cut antibiotic use, support sustainable aquaculture, and advance One Health goals; collaborative research will be key to translating these findings into practice.

## 1. Introduction

Aquaculture has emerged as an indispensable pillar of global protein production, underpinning food security and economic stability in an era of escalating population growth and diminishing wild fish stocks [1,2]. However, the sector’s rapid expansion is increasingly constrained by sustainability challenges, including recurrent disease outbreaks, antibiotic resistance, and environmental degradation [3,4]. Pathogens such as *Aeromonas hydrophila* and nervous necrosis virus (NNV) inflict severe mortality in farmed fish, incurring substantial economic losses [5,6], while the overuse of antibiotics to combat these threats has accelerated antimicrobial resistance, jeopardizing both aquaculture productivity and public health [3,4]. Compounding these issues, environmental stressors like microplastic contamination in shrimp ponds further destabilize production systems by inducing immune suppression and microbial dysbiosis in *Litopenaeus vannamei* [7,8]. Collectively, these challenges underscore the urgent need for innovative strategies that reconcile intensification with ecological and health imperatives.

Conventional disease management relies heavily on vaccines, yet their efficacy remains inconsistent across species and production environments. While inactivated vaccines are cost-effective, they frequently require adjuvants to enhance immunogenicity and often fail to confer durable protection [5,9]. For instance, oil-adjuvanted vaccines in Senegalese sole improved survival rates but did not fully prevent NNV infection, revealing gaps in adaptive immunity [5]. Practical limitations of delivery methods—oral, immersion, or injection—further impede progress, with issues ranging from mucosal antigen degradation to prohibitive production costs [9,10]. Environmental concerns, such as the ecological footprint of adjuvants, further complicate large-scale deployment [5]. These shortcomings emphasize the demand for sustainable alternatives that amplify vaccine performance while minimizing ecological risks.

Probiotics—live microorganisms conferring host health benefits—have gained prominence as immunostimulants and microbiome modulators in aquaculture. Strains like *Bacillus subtilis* and *Lactiplantibacillus plantarum* enhance mucosal immunity by upregulating IgM⁺ B cells, lysozyme activity, and cytokine expression [11,12]. Dietary co-culture of *Lactobacillus acidophilus* with *Bacillus subtilis* improved growth, immune-enzyme activities, and resistance to *Vibrio* infection in *Litopenaeus vannamei* fed low-fish-meal diets [13]. A *B. subtilis*-fermented *Caulerpa microphysa* by-product likewise served as a functional feed additive, elevating immune parameters and disease resistance in white shrimp [14]. Strain-level differences matter: fish-isolated *Bacillus velezensis* genotypes displayed distinct growth-promoting and cytokine-modulating profiles in zebrafish [15]. Plant-derived synbiotics are emerging too—*Lactobacillus plantarum*-fermented *Moringa oleifera* leaves boosted innate immunity and survival against *Aeromonas hydrophila* in Nile tilapia [16]. Beyond bacteria, antimicrobial peptides from scorpion venom simultaneously suppressed *Vibrio parahaemolyticus* and modulated shrimp immune genes [17]. Even fermented distillers’ dried grains with solubles (DDGS) have been shown to enrich beneficial gut taxa and enhance antioxidant status in zebrafish, indirectly supporting immune competence [18]. For instance, *B. subtilis* spores expressing viral VP2 protein elicited specific antibodies against infectious pancreatic necrosis virus (IPNV) in rainbow trout while retaining innate immunostimulatory properties [11]. Beyond direct immune activation, probiotics reshape gut microbiota, suppressing pathogens like *Achromobacter* while enriching beneficial taxa such as *Lactobacillus* and *Streptococcus* [19]. Synbiotic formulations—combining probiotics with prebiotics—further amplify immune responses and disease resistance in species ranging from shrimp to carp [12,20]. These multifaceted mechanisms position probiotics as versatile tools for priming host immunity and optimizing vaccine outcomes. This review posits that probiotics synergize with vaccines by enhancing mucosal barrier integrity, antigen presentation, and immune memory. Through microbiome modulation and immunostimulatory pathways, probiotics address critical limitations of conventional vaccines, offering a sustainable strategy to bolster aquaculture productivity and disease resilience.

## 2. Fish Immune System: Foundations for Vaccine Design

The teleost immune system integrates innate and adaptive components that collectively neutralize pathogens. Innate immunity delivers rapid, nonspecific defenses through phagocytes (e.g., macrophages, neutrophils) and antimicrobial peptides (AMPs), whereas adaptive immunity provides long-term protection via lymphocytes and immunoglobulins (Ig) [21,22]. Central to innate immunity are pattern recognition receptors (PRRs), including Toll-like receptors (TLRs), which identify pathogen-associated molecular patterns (PAMPs) and initiate cytokine production (e.g., TNF-α, IL-1β), thereby mobilizing immune cells [23,24]. For example, TLR5 and TLR9 in Atlantic salmon activate pro-inflammatory signaling cascades during bacterial infections [25]. In teleosts, adaptive immunity is orchestrated by T and B lymphocytes. CD8^+^ T cells in ginbuna carp, for instance, exhibit innate-like cytotoxicity against extracellular parasites through perforin and serine protease mechanisms [26]. B cells generate IgM, the dominant systemic antibody, alongside IgT, a key mediator of mucosal immunity [23]. While IgM levels correlate with post-vaccination pathogen clearance, teleosts lack lymph nodes, instead utilizing diffuse lymphoid tissues, such as the head kidney and spleen, for antigen presentation [27,28]. Recent work has expanded this view. The nurse shark pancreas was shown to function as a secondary lymphoid organ that supports antigen presentation and lymphocyte activation [29]. Proteomic profiling of chemically inactivated nodavirus-vaccinated European sea bass further revealed upregulation of redox and adaptive-immunity proteins, providing molecular signatures of vaccine-driven T- and B-cell activation [30]. Long-lived IgM⁺ memory B cells have now been demonstrated in common carp, persisting ≥ 6 months and differentiating into plasma cells on re-stimulation [31]. Cytokine-driven T-cell activation is also becoming clearer: interleukin-12 binding to its receptor promoted CD4⁺ Th1 differentiation and enhanced vaccine protection in flounder [32]. Ontogenetic studies in the near-threatened catfish *Clarias dussumieri* show early expression of MHC-IIβ and CD4 transcripts, marking maturation of cell-mediated immunity essential for successful vaccination [33]. Finally, the immersion adjuvant MONTANIDE IMS 1312 potentiated both innate and adaptive immunity in Nile tilapia, boosting specific IgM and T-cell–related cytokines after *Streptococcus agalactiae* vaccination [34]. Notably, cold-water species like Atlantic cod display diminished adaptive immune capabilities resulting from the loss of activation-induced cytidine deaminase (AID), highlighting evolutionary adaptations that compromise immune functionality [35].

Mucosal interfaces—skin, gills, and gut—constitute essential first-line defenses, hosting specialized immune cells and secretory IgT [23,36]. In gilthead sea bream, secretory IgZ (also called IgT), a teleost-specific immunoglobulin isotype analogous to mammalian IgA, within gill-associated lymphoid tissue (GALT), orchestrates mucosal immunity via the cGAS-STING pathway [23], whereas mucosal CD8^+^ T cells in ginbuna carp eradicate parasites through contact-dependent cytotoxicity [26,36]. However, developing effective mucosal vaccines remains challenging. Oral formulations frequently undergo degradation in the gastrointestinal tract, requiring nanoparticle carriers to safeguard antigens [37], whereas immersion vaccines demonstrate inconsistent efficacy owing to limited antigen penetration across epithelial barriers [38,39]. For instance, oral administration of formalin-killed *Vibrio* vaccines in turbot necessitates adjuvants to maintain IgM titers [24].

Current vaccine platforms include: (1) inactivated vaccines (e.g., formalin-killed *Aeromonas hydrophila*), which trigger IgM production but often require adjuvants for sustained efficacy; (2) live attenuated vaccines (LAVs), such as attenuated *Edwardsiella ictaluri* strains that boost phagocytic activity in catfish B cells; and (3) DNA/recombinant vaccines, exemplified by *Lactococcus lactis*-expressed *Vibrio* antigens that activate mucosal and systemic immunity through IL-12 and IFN-γ signaling [40,41,42,43]. Delivery methods profoundly influence outcomes: intraperitoneal injection induces robust systemic IgM responses but causes handling-related stress [44]; immersion facilitates mass vaccination yet frequently demands booster regimens [38]; and oral delivery enhances mucosal IgT production but requires microencapsulation to prevent enzymatic degradation [37,45].

Despite progress, critical challenges remain unresolved. Species-specific variability—such as diminished AID activity in Gadiformes—hinders antibody affinity maturation [35]. Proteolytic degradation of mucosal antigens in the gut demands innovative delivery platforms, while environmental stressors like hypoxia impair adaptive immunity, curtailing the duration of vaccine-mediated protection [37,46,47]. Furthermore, adjuvant reliance—illustrated by poly(I:C)’s requirement for temperature-controlled conditions to optimize interferon responses—emphasizes the necessity for customized formulations [39,48]. Overcoming these barriers necessitates strategies to improve antigen presentation, extend immune stimulation, and tailor responses to host specificity—advances that create opportunities for probiotic-driven immune modulation.

## 3. Probiotics in Aquaculture: Mechanisms and Impact

Probiotics in aquaculture are defined as live microorganisms that confer health benefits to aquatic hosts by enhancing growth, modulating immune responses, and improving pathogen resistance [49]. Commonly utilized genera include *Lactobacillus*, *Bacillus*, *Bifidobacterium*, and *Enterococcus*, selected based on safety (non-pathogenic, non-hemolytic), host-specific colonization capacity, and functional traits such as enzyme production (e.g., protease, amylase) or pathogen inhibition [50,51,52]. For example, *Bacillus subtilis* strains HGCC-1 and NTU-18 improve growth performance and gut health in golden pompano (*Trachinotus ovatus*) and grey mullet (*Mugil cephalus*), respectively, while *Lactococcus lactis* PH3-05 enhances larval survival and intestinal morphology in tropical gar (*Atractosteus tropicus*) [50,53,54]. Host-derived strains, such as *Bacillus amyloliquefaciens* AV5 isolated from Nile tilapia (*Oreochromis niloticus*), exhibit superior adaptation to host gut conditions, emphasizing the importance of strain specificity [55].

A pivotal mechanism of probiotics lies in their capacity to restore gut microbial balance by enriching beneficial taxa (e.g., *Lactobacillus*, *Bacillus*) while suppressing pathogens such as *Vibrio* and *Aeromonas* [56]. In *Epinephelus akaara*, the antimicrobial peptide Scy-hepc increased *Rhizobiaceae* abundance and reduced *Psychrobacter* colonization, enhancing intestinal barrier integrity via tight junction upregulation [57]. Similarly, *Bacillus subtilis* HGCC-1 elevated intestinal *Bacillus* populations and lipid metabolism-associated functional groups, alleviating hepatic steatosis in golden pompano [56]. Competitive exclusion is mediated through bacteriocin secretion and quorum-quenching enzymes; *Bacillus velezensis* B8, for example, inhibited *Aeromonas veronii* motility and biofilm formation in grass carp (*Ctenopharyngodon idella*) [58]. Probiotics also fortify mucosal defenses, as demonstrated by *Lactococcus lactis* PH3-05 upregulating muc-2 expression to enhance mucus production in tropical gar larvae [54].

Direct immunomodulation by probiotics involves the activation of innate immunity through enhanced phagocytosis, respiratory burst, and antioxidant enzyme production (Table 1) [52]. In Nile tilapia, *Bacillus safensis* NPUST1 elevated lysozyme activity and upregulated pro-inflammatory cytokines (IL-1β, TNF-α, IFN-γ), improving survival against *Streptococcus iniae* [59]. Metabolites such as short-chain fatty acids (SCFAs) from *Clostridium butyricum* enhance anti-inflammatory responses by promoting IL-10 secretion and mitigating oxidative stress [60]. Exopolysaccharides from *Lactobacillus plantarum* Ep-M17 boosted IgM titers and complemented C3 expression in *Litopenaeus vannamei*, illustrating adaptive immune priming [61]. Probiotic-derived bile acids, such as taurocholic acid (TCA) and glycochenodeoxycholic acid (GCDCA), regulate lipid metabolism and immune homeostasis in zebrafish (Danio rerio) via farnesoid X receptor (FXR) signaling [62]. These immunomodulatory effects synergize with vaccines; for example, *Bacillus subtilis* spores expressing *Vibrio harveyi* FlgE elevated IgM production and reduced *Vibrio* colonization in grouper (*Epinephelus* spp.) [63].

Probiotic efficacy is influenced by environmental and host factors, including water quality, diet composition, and host genetics. High stocking density in Nile tilapia reduced the effectiveness of *Bacillus amyloliquefaciens* AV5, necessitating higher doses to counteract oxidative stress [55]. Diet prebiotics (e.g., sodium gluconate) amplified *Bacillus velezensis* R-71003’s antioxidant and TLR4-mediated immune responses in common carp (*Cyprinus carpio*) [64]. Host specificity is critical, as demonstrated by *Lactobacillus plantarum* Ep-M17, derived from grouper, which exhibited superior colonization and immune activation in shrimp compared to non-host strains [61]. Environmental contaminants, such as polystyrene nanoplastics (PS-NPs), disrupted *Bacillus subtilis*-mediated immune enhancement in grass carp, highlighting the need for adaptive formulations under pollution stress [56]. Collectively, these probiotic functions—from direct pathogen suppression and competitive exclusion to targeted immune priming and microbiome remodeling—pave the way for integrating live microbial adjuvants with vaccination strategies to optimize protective outcomes.

**Table 1:** Mechanisms of probiotic-driven vaccine enhancement in fish aquaculture.

Mechanism	Probiotic Example(s)	Immunological Effects	Impact on Vaccine Efficacy	Sustainability Benefit(s)	Key References
Antigen protection and delivery	*Bacillus subtilis spores* (expressing FlgE)	Enhances mucosal IgT production in grouper; shields antigens from degradation.	↑ Antigen persistence in gut → prolonged immune activation.	Reduces vaccine doses; minimizes ecological disruption.	[63]
Immune priming	*Lactobacillus plantarum* Ep-M17	↑ Phenoloxidase, lysozyme activity; upregulates proPO and pen4 in white shrimp.	Strengthens innate-to-adaptive immunity transition.	Reduces antibiotic reliance via enhanced pathogen resistance.	[65]
Microbiome modulation	*Bacillus velezensis* FIO1408	Suppresses *Vibrio* spp.; enriches beneficial gut microbiota in Asian seabass.	↓ Pathogen competition → improves vaccine-targeted responses.	Promotes closed-loop systems; reduces waste.	[66]
Cross-mucosal immunity	*Lactobacillus pentosus* BD6	Induces IgT+ B cells in skin/gills of white shrimp; enhances intestinal microbiota diversity.	Broad-spectrum protection across mucosal and systemic sites.	Reduces need for species-specific vaccines.	[67]
Quorum quenching	*Bacillus* spp. (e.g., *B. subtilis* RODK2810C3)	Degrades AHLs; inhibits *Edwardsiella tarda* virulence in zebrafish and rohu.	Enhances vaccine efficacy by weakening pathogen virulence.	Lowers antibiotic use; eco-friendly disease control.	[68]
Eco-friendly formulations	*Phaeobacter piscinae* S26 (TDA-producing)	Antagonizes *Vibrio* coralliilyticus in fish larvae without antibiotic resistance risks.	Comparable efficacy to antibiotics in pathogen suppression.	Minimizes chemical inputs; safe for marine ecosystems.	[69]
Competitive exclusion	*Streptomyces* sp. SH5	Reduces *Aeromonas hydrophila* colonization in zebrafish via biofilm inhibition.	↓ Pathogen adhesion → enhances vaccine-specific responses.	Reduces disease outbreaks in closed systems.	[70]
Synbiotic synergy	*Bacillus* sp. PM8313 + red sea bream	Improves digestive enzymes and immune genes microbiota (e.g., SOD, CAT) in red sea bream.	Enhances nutrient absorption → supports vaccine-induced immunity.	Reduces feed waste; improves growth efficiency.	[71]
Pathogen inhibition	*Weissella confusa N17*	Inhibits *Aeromonas veronii* in loach via adhesion competition and biofilm disruption.	Protects mucosal surfaces → strengthens vaccine efficacy.	Reduces chemical treatments; promotes host-specific probiotics.	[72]

Note: IgT: immunoglobulin T; AHLs: acyl homoserine lactones; TDA: tropodithietic Acid; SOD: superoxide dismutase; CAT: catalase; ProPO: prophenoloxidase; Pen4: penaeidin 4; *Vibrio* spp.: *Vibrio species*; ↑: increase; ↓: decrease; →: change or transition.

## 4. Probiotic–Vaccine Synergy: Evidence and Mechanisms

Probiotics have emerged as potent adjuvants in aquaculture vaccination strategies, enhancing antigen uptake and prolonging immune exposure through mucosal and systemic pathways. Engineered strains such as *Escherichia coli Nissle 1917* exemplify this role, delivering antigens (e.g., tumor antigens or β-glucan) to augment macrophage and dendritic cell phagocytosis, thereby priming adaptive immunity [73]. Probiotics like *Lactobacillus casei* and *Bacillus subtilis* further amplify immune responses by activating Toll-like receptors (TLRs; e.g., TLR4), increasing antigen-presenting cell (APC) activity and cytokine production [74,75]. The synergy between probiotics and vaccines operates through three primary mechanisms: (1) antigen delivery, where probiotics serve as carriers for pathogen-specific antigens (e.g., *Saccharomyces cerevisiae* displaying the white spot syndrome virus VP28), enhancing mucosal IgA and systemic IgG responses [76]; (2) immune priming, wherein probiotics induce trained immunity—a form of enhanced innate immune memory created through epigenetic and metabolic reprogramming of macrophages and other innate cells—promoting long-term antigen-specific T-cell activation [73,77]; and (3) microbiome modulation, through which probiotics suppress pathogenic *Vibrio* spp. colonization while enriching beneficial taxa, thereby enhancing mucosal barrier integrity [63].

Experimental comparisons of probiotic pre-treatment and co-administration reveal distinct immunological outcomes. Pre-treatment with *Lactobacillus rhamnosus*-fermented *Acanthopanax senticosus* for eight weeks in crucian carp elevated serum IgM, lysozyme activity, and survival rates (60%) following *Aeromonas hydrophila* challenge [78]. Similarly, co-administration of *Bacillus subtilis* spores displaying *Vibrio* OmpK antigen boosted European seabass survival by 86% against *V. anguillarum*, accompanied by anti-OmpK antibodies [75]. Case studies further validate this synergy: recombinant *Lactobacillus casei* expressing *Aeromonas Aha1* antigen stimulated serum IgM, phagocytosis, and 60% survival in common carp [79], while *B. subtilis* spores expressing *Vibrio* OmpK induced 90% survival in zebrafish challenged with *V. parahaemolyticus* [75].

**Figure 1:**
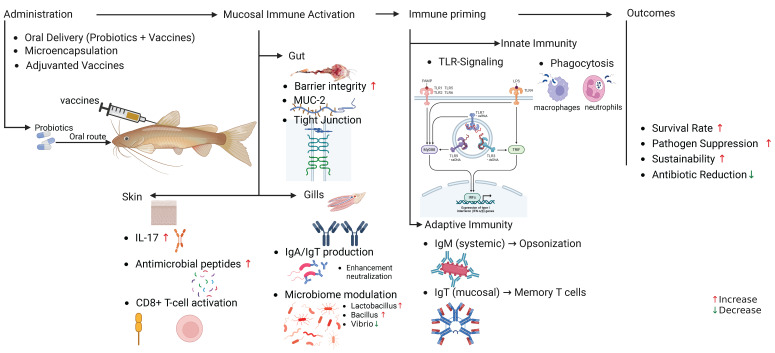
Mechanistic overview of probiotic–vaccine synergy in fish mucosal immunity. This schematic illustrates the interplay between probiotics (e.g., *Bacillus subtilis*, *Lactobacillus casei*) and vaccines in enhancing mucosal and systemic immunity. Key pathways include (1) antigen protection via probiotic encapsulation (e.g., chitosan–alginate microcapsules); (2) immune priming through toll-like receptor (TLR) activation and cytokine production (e.g., IL-1β, TNF-α); (3) microbiome modulation, suppressing pathogens (*Vibrio* spp.) while enriching beneficial taxa (*Lactobacillus*); and (4) cross-mucosal activation of IgT^+^ B cells in the gut, skin, and gills.

Mechanistic insights highlight four interconnected pathways: (1) APC activation, where probiotics like *Bacillus subtilis* enhance dendritic cell recruitment and MHC-II expression, facilitating antigen cross-presentation to CD4^+^ and CD8^+^ T cells [11]; (2) antigen persistence, achieved via amphiphilic exopolysaccharides (e.g., NAPS) that self-assemble with antigens to prolong pharmacokinetics and IgG titers [74]; (3) gut-systemic immunity cross-talk, exemplified by *Lactobacillus plantarum* upregulating intestinal IL-1β, TNF-α, and IFN-γ, which correlate with systemic increases in IgM and complement proteins [56]; and (4) microbiome modulation, wherein *Bacillus subtilis* reduces pathogenic *Vibrio* abundance in grouper intestines while enriching beneficial *Bacillus* spp., amplifying vaccine-induced immunity [63]. Heat-killed *Lactobacillus rhamnosus* GG (HK-LGG) exemplifies immune priming, stimulating dendritic cells to secrete IL-23, which activates type 3 innate lymphoid cells (ILC3s) and IL-22 production to fortify mucosal barriers [80]. Collectively, these mechanisms position probiotics as dual-purpose tools—antigen carriers and immune modulators—that bridge innate and adaptive responses to optimize vaccine efficacy (Figure 1).

## 5. Mucosal Immunity and Oral Vaccination: Probiotics as Game-Changers

Oral vaccination in fish aquaculture is hindered by three primary challenges: antigen degradation in the gastrointestinal tract, poor mucosal antigen uptake due to intestinal immune tolerance, and insufficient stimulation of localized immunity [81,82]. For instance, unprotected DNA vaccines in Senegalese sole yielded a mere 6.25% relative percent survival (RPS), post-challenge, attributed to rapid enzymatic degradation [82]. Similarly, oral vaccines employing non-encapsulated antigens often fail to elicit robust humoral or cellular responses, underscoring the need for advanced delivery systems that protect antigens and enhance immunogenicity [81].

Probiotics such as *Bacillus subtilis* and *Lactobacillus casei* offer innovative solutions by enhancing gut integrity and modulating mucosal immunity. Engineered *B. subtilis* spores displaying viral antigens protect against degradation while stimulating adaptive immunity, as demonstrated in grass carp vaccinated against grass carp reovirus (GCRV), which exhibited upregulated IgM expression and increased survival [83]. Probiotics interact with mucosal immune cells, including dendritic cells and IgT+ B cells, to improve antigen presentation and bridge mucosal-systemic immunity (Figure 1). For example, *B. subtilis* strains upregulated genes associated with inflammation and T-cell responses in rainbow trout, enhancing both innate and adaptive defenses [11]. Recombinant *L. casei* expressing *Aeromonas veronii* MshB antigen elevated serum IgM and mucosal IgA levels in crucian carp, illustrating synchronized immune responses across compartments [84].

Notable advancements include chitosan–alginate microcapsules encapsulating *Lactobacillus rhamnosus* expressing koi herpesvirus (KHV) ORF81 protein. This probiotic-based oral vaccine achieved an 85% survival rate in carp by protecting antigens from gastric degradation and stimulating neutralizing IgM antibodies [85]. Similarly, germination-arrested *B. subtilis* spores delivering GCRV VP7 antigen enhanced humoral and cellular immunity in grass carp, outperforming the efficacy of traditional vaccines [57]. These examples underscore how probiotics synergize with oral vaccines to overcome delivery barriers and amplify protective immunity.

Beyond intestinal immunity, probiotics show promise in enhancing protection at other mucosal interfaces, such as the skin and gills. Oral administration of *L. casei* expressing *Edwardsiella tarda* antigens in olive flounder elevated serum and skin mucus IgM titers, indicating cross-mucosal immune activation [86]. Probiotic-adjuvanted oral vaccines in Nile tilapia induced antigen-specific IgM in both serum and mucus, suggesting systemic and localized responses [87]. Although direct evidence for gill immunity remains limited, probiotics’ broad immunomodulatory effects—such as upregulated lysozyme activity in skin mucus—suggest potential for reinforcing external mucosal defenses [82]. Future vaccine designs could leverage probiotics to target multiple mucosal sites, offering comprehensive disease resistance while minimizing invasive delivery methods.

## 6. Innovations in Probiotic-Based Adjuvants

Traditional adjuvants, including aluminum hydroxide and glucans, remain staples in vaccine formulation but are constrained by safety concerns, limited specificity, and poor mucosal targeting [6]. For instance, while aluminum hydroxide improved survival rates in *Aeromonas veronii*-challenged crucian carp, its efficacy was surpassed by flagellin-based adjuvants, which achieved a 78.37% survival rate [88]. However, such adjuvants often induce unintended inflammatory responses and lack mechanisms to protect antigens in harsh mucosal environments [6]. In contrast, probiotic-based adjuvants offer inherent biocompatibility, mucosal adherence, and dual functionality as antigen carriers and immune modulators. *Bacillus subtilis* spores exemplify this dual functionality, protecting antigens from gastrointestinal degradation while enhancing adaptive immunity in grass carp (*Ctenopharyngodon idella*) and outperforming traditional delivery systems [57]. Similarly, *Lactobacillus plantarum* induces secretory IgA (sIgA) and T-cell responses, demonstrating cross-species versatility [89].

Advances in genetic engineering have transformed probiotics into precision antigen-delivery platforms. Surface-display systems, such as *Bacillus subtilis* spores engineered with CotB/CotC proteins to anchor grass carp reovirus (GCRV) VP7 antigen, prevent antigen loss during germination and significantly improve protection rates [57]. Recombinant *Lactobacillus casei* expressing *Vibrio* mimicus OmpU fused to cholera toxin B subunit (CTB) elicited robust IgM and cytokine responses in *Carassius auratus*, achieving 58.33% survival post-challenge [90]. Controlled-release systems, including biofilm matrices, are exemplified by *Lactobacillus plantarum* displaying Singapore grouper iridovirus (SGIV) VP19 on spores, which colonized the intestine and reduced viral loads by 28.7% in grouper [91]. Engineered *Bacillus subtilis* expressing Vibrio harveyi FlgE protein not only enhanced survival (63% RPS) but also suppressed pathogenic *Vibrio* abundance through gut microbiota modulation [63].

Synbiotic formulations—combining probiotics with prebiotics—amplify the viability and functionality of engineered strains (Table 2). For example, *Bacillus coagulans* synergized with recombinant *Lactobacillus casei* expressing *Aeromonas veronii* MshB, elevating mucosal immunity and survival rates in carp [84]. Nanotechnology further refines delivery: chitosan–alginate microcapsules protected *Lactobacillus rhamnosus* vaccines against gastric degradation, achieving 85% protection in koi carp against herpesvirus [85], while polylactic acid (PLA) microspheres encapsulating *Lactobacillus casei* expressing largemouth bass virus (LMBV) antigens improved intestinal colonization and survival rates from 24% to 68% [92]. Nanoparticle co-delivery systems, which simultaneously transport antigens and probiotics, enhance stability and targeted immune activation, bridging lab-scale innovations to scalable aquaculture solutions [6].

Emerging CRISPR-Cas technologies enable precise genetic modifications to optimize probiotic adjuvant functionality. CRISPR interference (CRISPRi) applied to marine bacteria like *Pseudoalteromonas luteoviolacea* modulates host-microbe interactions and secondary metabolite production, offering tailored immune modulation [93]. CRISPR-edited *Lactobacillus* strains engineered to express pathogen-specific antigens (e.g., viral VP28 or bacterial OmpK) act as dual-purpose adjuvants, delivering antigens while stimulating mucosal immunity via innate pathways such as TLR signaling [94]. Recent work has demonstrated an inducible two-plasmid CRISPR/Cas9 system for chromosomal gene insertion in *Lactiplantibacillus plantarum* WCFS1, enabling efficient knock-in of antigen and reporter cassettes to streamline probiotic vaccine delivery [94]. CRISPR/Cas9-mediated immune modulation in fish, such as IgM knockout in salmon, has also shown promise for studying adaptive immune responses [95]. Additionally, CRISPR-edited probiotics can be programmed to secrete immunomodulatory metabolites (e.g., short-chain fatty acids) or surface-display antigens, synergizing with oral or immersion vaccines [96]. While challenges such as strain-specific optimization and regulatory hurdles persist, CRISPR technologies offer a transformative pathway to develop next-generation probiotics that enhance vaccine efficacy and sustainability.

**Table 2:** Probiotic–vaccine synergy studies in fish aquaculture.

Probiotic Strain	Antigen/Adjuvant	Delivery Method	Target Pathogen	Host Species	Survival Rate/Protection	Key Immune Outcomes	Reference
*Lactobacillus casei*	MCP2α (LMBV) + FlaC (adjuvant)	PLA microspheres (oral)	Largemouth bass ranavirus (LMBV)	Largemouth bass (Micropterus salmoides)	↑ Survival: 24% → 68%	↑ Serum enzymes (T-SOD, LZM, C3); ↑ immune genes (IL-1β, TNF-α, IFN-γ); reduced viral load	[92]
*Lactobacillus casei*	Aha1 (*Aeromonas veronii*) + CTB adjuvant	Oral (surface-displayed)	*Aeromonas veronii*	Common carp (Cyprinus carpio)	↑ Survival: 53.57% → 64.29%	↑ IgM, ACP, AKP, SOD; ↑ cytokines (IL-1β, TNF-α); intestinal colonization	[79]
*Lactobacillus casei*	Aha1 (*Aeromonas hydrophila*)	Oral (recombinant vectors)	*Aeromonas hydrophila*	Common carp	↑ Survival: 50% → 60%	↑ IgM, AKP, SOD; ↑ cytokines (IL-1β, TNF-α); reduced bacterial load	[79]
*Lactobacillus casei*	Aha1 + LTB adjuvant	Oral (surface-displayed)	*Aeromonas veronii*	Carp	↑ Survival: 53.57% → 60.71%	↑ IgM, LZM, C3; ↑ cytokines (IL-1β, TNF-α); reduced tissue damage	[97]
*Lactobacillus casei*	MshB (*Aeromonas veronii*) + CTB adjuvant	Oral	*Aeromonas veronii*	Crucian carp (*Carassius auratus*)	↑ Survival: 48% → 60%	↑ IgM, SOD, C3; ↑ cytokines (IL-1β, TNF-α); reduced bacterial load	[98]
*Lactobacillus rhamnosus*	Fermented *Acanthopanax senticosus*	Oral	*Aeromonas hydrophila*	Crucian carp	↑ Survival: 40% → 60%	↑ Antioxidants (CAT, SOD); ↑ cytokines (IL-1β, TNF-α); reduced tissue pathology	[78]

Note: PLA: poly(lactic acid); MCP2α: major capsid protein 2 alpha; LMBV: largemouth bass ranavirus; IgM: immunoglobulin M; LZM: lysozyme; C3: complement component 3; SOD: superoxide dismutase; IL-1β: interleukin 1 beta; TNF-α: tumor necrosis factor alpha; IFN-γ: interferon gamma; CTB: cholera toxin B subunit; AKP: alkaline phosphatase; ACP: acid phosphatase; ↑: increase; →: change or transition.

## 7. Challenges and Research Gaps

The application of probiotics in aquaculture faces significant scientific and practical hurdles, beginning with strain-specific variability in efficacy and mechanisms (Table 1). Studies employing diverse species such as *Bacillus subtilis*, *Lactococcus lactis*, and *Shewanella algae* report inconsistent outcomes. For instance, *Bacillus cereus* SL1 synergized with *Ocimum sanctum* to enhance immunity in *Cirrhinus mrigala*, while *Paecilomyces variotii* modulated immune biomarkers in Atlantic salmon pre-smolts in a dose-dependent manner [99,100]. Such variability underscores the need for standardized protocols to evaluate strain-host compatibility, particularly for oral delivery systems. While chitosan–alginate microcapsules improve vaccine viability in carps, production parameters such as bioreactor conditions require optimization to ensure consistency [85,101]. Emerging machine learning (ML) approaches, such as Bayesian networks (e.g., SAMBA), could predict strain efficacy and optimize delivery systems, though their application remains nascent [102].

A critical limitation lies in the lack of multi-omics data to elucidate host-microbiome-immune interactions. Metagenomic analyses identified core intestinal bacteria in *Litopenaeus vannamei* and linked abundance of representatives of the family *Rhodobacteraceae* to immune gene upregulation [103,104], yet few studies integrate transcriptomics or proteomics. For example, shrimp transcriptomics revealed correlations between *the presence of Rhodobacteraceae* species and NF-κB signaling but did not explore how probiotic-induced microbial shifts alter systemic immune responses [103]. Notably, enrichment of pathogenic *Photobacterium* strains—linked to opportunistic infections under dysbiotic conditions—highlights the need for strain-level risk assessment in probiotic applications [105]. Integrating metabolomics and ML models could bridge this gap, mapping microbial dynamics to immune pathways, thus enabling targeted probiotic therapies [86].

Practical barriers include cost, scalability, and farmer education. While oral probiotics encapsulated in chitosan–alginate systems offer farm-level feasibility, small-scale farmers often lack training on optimal dosing. In Egypt, only 30% of tilapia farmers adhered to recommended probiotic protocols without workshops, resulting in suboptimal growth [106]. Low-cost viability assays (e.g., ATP bioluminescence kits) and AI-driven tools could empower farmers to monitor probiotic quality. Regulatory concerns further complicate adoption. The EU’s three-year evaluation of *Bacillus subtilis* as a feed additive delayed approvals despite prior validation, highlighting the need for streamlined frameworks [107]. CRISPR-edited probiotics with “kill switches” or virulence gene knockouts may expedite approvals by addressing biosafety risks [93].

Unanswered questions persist regarding the long-term stability of probiotic-induced microbiome shifts post-vaccination. *Bacillus subtilis* spores transiently reduced *Vibrio* abundance in grouper intestines but showed limited persistence, suggesting staggered dosing or metabolite supplementation (e.g., salicylaldehyde) to sustain microbial resilience [63,108]. Optimal timing and dosage for probiotic–vaccine synergy remain unclear; while Ecobiol Plus^®^ at 0.4 g/kg improved disease resistance in Nile tilapia, interactions between probiotic administration and vaccination schedules were unexplored [106]. Pre-treatment with *Lactobacillus* to prime TLR pathways before vaccination could enhance antigen uptake, though empirical validation is needed [11].

Ecological risks, such as the enrichment of pathogenic *Photobacterium* strains linked to opportunistic infections in salmon guts following probiotic use, warrant investigation to ensure environmental safety [100]. Synbiotic formulations (e.g., *Bacillus coagulans* + prebiotics) may mitigate spillover risks while amplifying mucosal immunity [84]. Addressing these challenges requires a holistic approach, balancing efficacy, safety, and scalability to realize the full potential of probiotic–vaccine strategies in sustainable aquaculture.

## 8. Future Directions

The integration of probiotics with vaccines holds transformative potential for aquaculture disease management, yet unlocking this synergy requires strategic innovation and interdisciplinary collaboration. Emerging evidence highlights the promise of early-life interventions, where probiotic and vaccine regimens prime immune systems during critical developmental windows. Dietary supplementation with *Bacillus subtilis* in zebrafish (*Danio rerio*) enhanced intestinal barrier integrity and upregulated immune-related genes (zo-1, occludin), suggesting larval-stage probiotics could establish lifelong immune resilience [109]. Similarly, *Lactobacillus acidophilus* AC improved antioxidant capacity and survival in juvenile zebrafish, demonstrating the feasibility of integrating probiotics into hatchery feeds to bolster innate immunity before pathogen exposure [110]. Future protocols could combine these early interventions with mucosal vaccines, such as codon-deoptimized mRNA vaccines against white spot syndrome virus (WSSV), which have shown enhanced safety and immune activation in shrimp [111].

The efficacy of probiotic–vaccine combinations hinges on species- and environment-specific customization. Autochthonous probiotics, such as *Bacillus amyloliquefaciens* COFCAU_P1 isolated from *Labeo rohita*, exhibit superior adhesion to host mucus and pathogen inhibition compared to allochthonous strains, underscoring the necessity for tailored probiotic selection [112]. Likewise, *Shewanella algae* and *Vibrio diabolicus*, identified as indigenous probiotics in *Litopenaeus vannamei*, demonstrated strain-specific benefits in pathogen resistance and microbiota modulation [104]. Advances in metagenomic screening and host-microbiome profiling will enable precise matching of probiotics to target species and farming conditions, optimizing synergistic effects with vaccines.

Probiotic–vaccine strategies are pivotal for reducing antibiotic dependence and advancing circular aquaculture systems. For example, *Clostridium butyricum* supplementation allowed partial replacement of fishmeal with cottonseed protein concentrate in shrimp diets, mitigating waste while enhancing immunity [113]. *Bacillus velezensis* BV1704-Y, derived from aquaculture waste, improved disease resistance in zebrafish by modulating gut microbiota and reducing pro-inflammatory cytokines [110]. Future systems could leverage fish processing byproducts as substrates for cultivating autochthonous probiotics through microbial fermentation or enzymatic hydrolysis. Fermentation of pangasius waste with a microbial consortium (*Pseudomonas aeruginosa*, *Rhizopus microsporus*, *Yarrowia lipolytica*) increased crude protein content by 37.27%, indicating its potential as a nutrient-rich probiotic source [114]. Enzymatic hydrolysis of seafood waste using alkaline proteases yielded bioactive peptides that supported *Bacillus* spp. growth, further aligning with circular bioeconomy principles [115,116]. Heat-killed probiotics, such as *Lactobacillus acidophilus* HLA, offer sustainable alternatives with reduced regulatory hurdles, achieving comparable efficacy to live strains in enhancing antioxidant and anti-inflammatory responses [109].

Accelerating probiotic–vaccine innovation requires collaboration across aquaculture, immunology, and data science. Transcriptomic analyses of vaccinated Atlantic salmon identified key immune pathways (e.g., TLR and IFN signaling), which could be amplified by probiotic co-administration [100]. Machine learning models analyzing gut microbiota datasets from Nile tilapia fed *Bacillus subtilis* natto NTU-18 could predict optimal probiotic–vaccine pairings [117]. Partnerships between academia and industry will be vital to translate lab-scale findings into scalable solutions, such as nanoparticle-based oral vaccines combined with probiotics for targeted mucosal delivery [118].

## 9. Conclusions

Probiotic–vaccine synergy represents a paradigm shift in aquaculture disease management, addressing critical limitations of conventional vaccines by enhancing mucosal immunity through pathogen exclusion, barrier fortification, and IgA/IgT stimulation. Probiotics such as *Bacillus subtilis* and *Lactobacillus casei* amplify antigen uptake and prolong immune exposure while modulating microbiomes to suppress opportunistic pathogens (e.g., *Vibrio* spp.) and enrich beneficial taxa like *Lactobacillus*, fostering a resilient gut ecosystem. Experimental successes—such as 86% survival in *Vibrio*-challenged European seabass via *B. subtilis*-OmpK vaccines—underscore the transformative potential of these strategies. Innovations in antigen-displaying spores, synbiotics, and nanotechnology resolve oral vaccine degradation and delivery inefficiencies, bridging lab research to field applications. Realizing these potential demands urgent collaboration across academia, industry, and policymakers. Academia must prioritize translational studies validating probiotic–vaccine efficacy under diverse farming conditions, while the industry invests in scalable production of engineered probiotics and delivery systems. Policymakers play a pivotal role in streamlining regulatory frameworks to fast-track approvals for probiotic-adjuvanted vaccines, ensuring safety without stifling innovation. Funding initiatives should target clinical trials, farmer education programs, and microbiome monitoring tools to build stakeholder confidence. Looking ahead, a microbiome-informed aquaculture industry is not only feasible but essential under the One Health framework. By replacing antibiotics with probiotic–vaccine synergies, aquaculture can reduce environmental contamination, curb antimicrobial resistance, and enhance food security. Integrating hatchery priming, personalized formulations, and circular bioeconomy principles will foster systems where fish health, ecosystem balance, and human well-being converge. This vision calls for global commitment to reimagine aquaculture as a pillar of planetary health, where science, sustainability, and stewardship nourish future generations.

## Data Availability

No new data were created or analyzed in this study. Data sharing is not applicable to this article.
